# Association of Medical Editors

**Published:** 1881-07-20

**Authors:** 


### *ASSOCIATION OF MEDICAL EDITORS.*

"This body of medical journalists, which meets during the session of the American Medical Association, held their annual meeting at the Exchange Hotel, Richmond. In the absenee of the president, Dr. J. F. Shrady, Dr. J. A. Octerlony, of Kentucky, was called to the chair, Dr. D. S. Reynolds, of Kentucky, acting as secretary.

The president's address was read by Dr. Carpenter, and ordered to be printed.

The committee on Necrology (Drs. Dunster, Cole and Edwards) reported the demise during the past year of two members (Drs. Davis and Cowling). The committee was allowed time to complete the memoirs.

The committee appointed to suggest officers for the ensuing year, named the following, which were confirmed:

For President—Dr. Landon B. Edwards, of Richmond, Va.

Vice-President—Dr. Ralph Walsh, of Washington, D. C.

Secretary—Dr. D. S. Reynolds, of Kentucky.

After an interchange of views the Association adjourned to meet on the Monday evening preceding the next annual meeting of the American Medical Association."

That the Editors' Association is not duly appreciated, we have heretofore stated. We regard it, if properly conducted, as a highly important body. In another place we refer to the agency of journalism in the elevation of Medical Education. The members of the profession throughout the country, more than by any other agency, are influenced by the journals they read, and a wholesome medical literature is of the utmost importance. The Association of Editors should look to the encouragement among themselves of a high moral and social brotherhood, free from envies and jealousies, and liberal in all the courtesies of journalism. Among the leading principles and objects upon which there should be the most perfect unanimity are the promotion of medical progress, the elevation of the standard of medical education, the encouragement of a high ethical standard, and the principles of truth, integrity and honor in the ranks of the profession.

